# Intestinal Obstruction Caused by Ascaris lumbricoides in a Pediatric Patient: A Case Report and Literature Review

**DOI:** 10.7759/cureus.98047

**Published:** 2025-11-28

**Authors:** Gloria Barrera Sánchez, Yunuén Maqueda Sánchez, Raúl Amílcar Cetina Castillo

**Affiliations:** 1 General Surgery, Hospital General de Cancún “Dr. Jesús Kumate Rodríguez”, Servicios de Salud Instituto Mexicano del Seguro Social (IMSS) Bienestar, Cancún, MEX; 2 Faculty of Medicine, Universidad Autónoma de Yucatán, Mérida, MEX

**Keywords:** amebiasis infection, enterotomy, helminth infection, intestinal obstruction, intestinal parasite, pediatric surgery, round worm

## Abstract

Ascariasis is one of the most common intestinal helminth infections worldwide, particularly in tropical regions with poor sanitary conditions. Although often asymptomatic, a heavy worm burden can cause severe complications such as intestinal obstruction. We present the case of an eight-year-old female child from a rural area of Quintana Roo, Mexico, who developed abdominal pain, vomiting, and absence of bowel movements. Computed tomography suggested intestinal obstruction with free peritoneal fluid and mural thickening. Emergency laparotomy revealed a conglomerate of *Ascaris lumbricoides* causing complete obstruction of the proximal ileum; enterotomy and worm extraction were performed successfully. The patient recovered well and was discharged on postoperative albendazole and ivermectin. This case underscores the need to consider ascariasis in the differential diagnosis of intestinal obstruction in children from endemic regions and highlights the importance of timely surgical intervention alongside antiparasitic therapy.

## Introduction

Ascariasis remains one of the most common helminthic infections worldwide, particularly in low-resource and tropical regions, where sanitation limitations facilitate fecal-oral transmission [1,2]. Children are disproportionately affected due to frequent soil exposure, inadequate hygiene practices, and a narrower intestinal lumen that predisposes them to mechanical complications when parasite burden is high [2]. Although most infections are asymptomatic, heavy infestation can lead to severe manifestations, including intestinal obstruction, one of the most clinically significant complications due to its potential for rapid deterioration.

Early recognition is essential because obstructive symptoms caused by *Ascaris lumbricoides* often mimic other parasitic and non-parasitic abdominal diseases [1]. Imaging may suggest obstruction but frequently fails to visualize the worm bolus directly. Highlighting these diagnostic challenges is particularly relevant in endemic settings, where advanced imaging or pediatric endoscopy may not be available. This case emphasizes the need for heightened clinical suspicion, especially in children from areas with poor sanitation or inconsistent access to routine deworming programs.

## Case presentation

An eight-year-old female child from Quintana Roo, Mexico, with no significant past medical history, presented with a three-day history of abdominal pain initially localized to the mesogastrium and later generalized. This was associated with multiple episodes of vomiting and absence of bowel movements. No fever was reported. She was initially managed at a local health center with enemas, without improvement, and was subsequently referred to our hospital.

On admission, the patient was conscious and oriented, with mild dehydration. Vital signs were: heart rate 102 beats per minute, respiratory rate 22 breaths per minute, blood pressure 87/47 mmHg, and temperature 36°C. Physical examination revealed a mildly distended abdomen with decreased peristalsis, tympanic sounds in all quadrants, diffuse tenderness more pronounced in the lower abdomen, and rebound tenderness. Laboratory results showed leukocytosis with neutrophilia and elevated C-reactive protein. A summary of results is presented in Table 1.

**Table 1:** Laboratory results on admission

Parameter	Patient Value	Reference Range
Hemoglobin (g/dL)	15.1	11.5–15.5
Hematocrit (%)	42.7	35–45
White blood cells (/µL)	16,100	4,000–11,000
Neutrophils (%)	81.8	40–70
Eosinophils (%)	7.5	1–6
Platelets (/µL)	404,000	150,000–450,000
C-reactive protein (mg/L)	26.8	<5
Sodium (mmol/L)	126	135–145
Potassium (mmol/L)	4.1	3.5–5.0
Creatinine (mg/dL)	0.3	0.5–1.0

**Figure 1:**
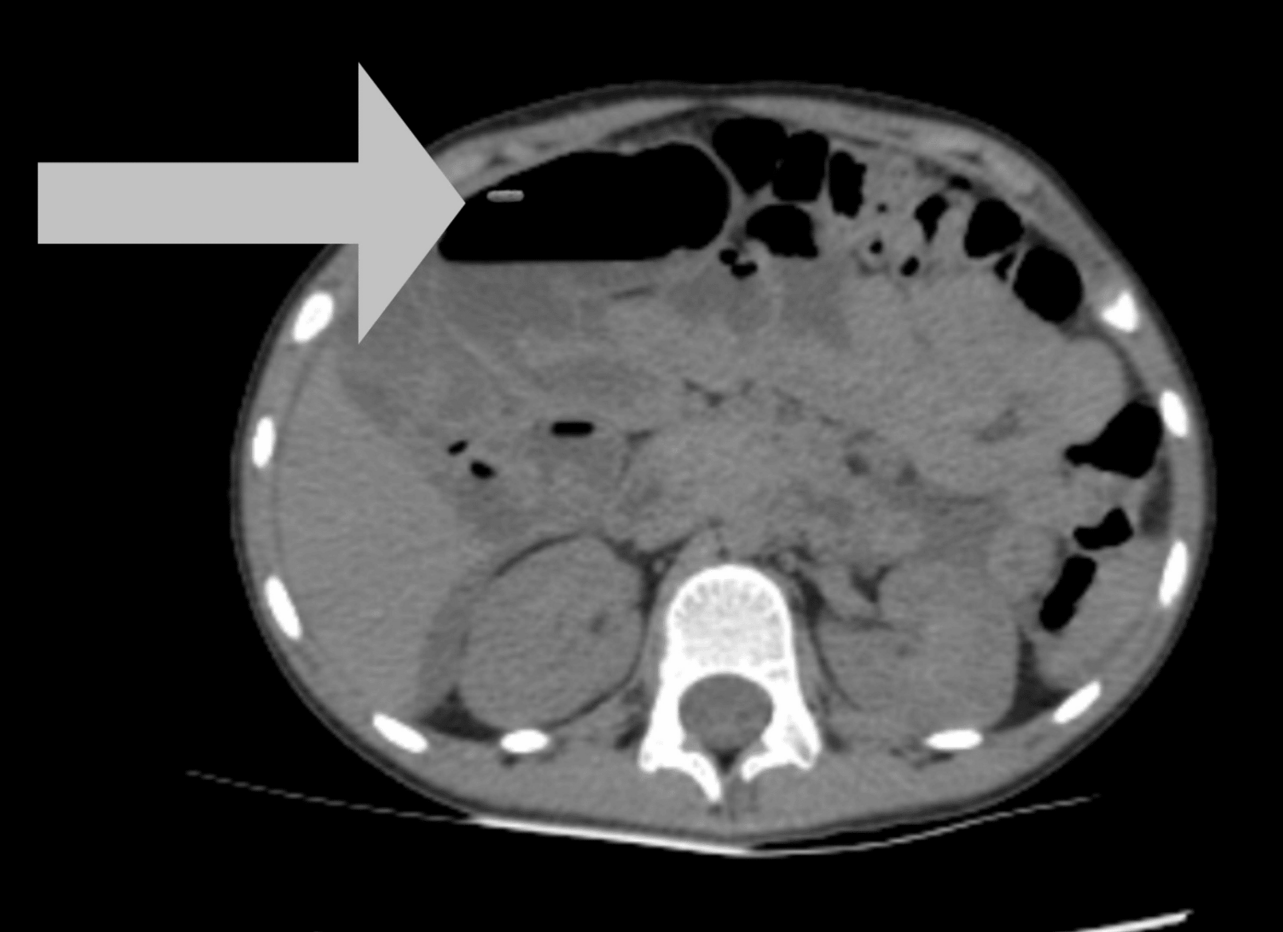
Abdominal computed tomography showing generalized colonic dilation with mural thickening of the sigmoid (arrow) and free peritoneal fluid, suggestive of intestinal obstruction.

Abdominal computed tomography demonstrated generalized colonic dilation with air-fluid levels, mural thickening of the sigmoid, and abundant free peritoneal fluid, findings suggestive of intestinal obstruction with possible volvulus (Figure 1).

**Figure 2:**
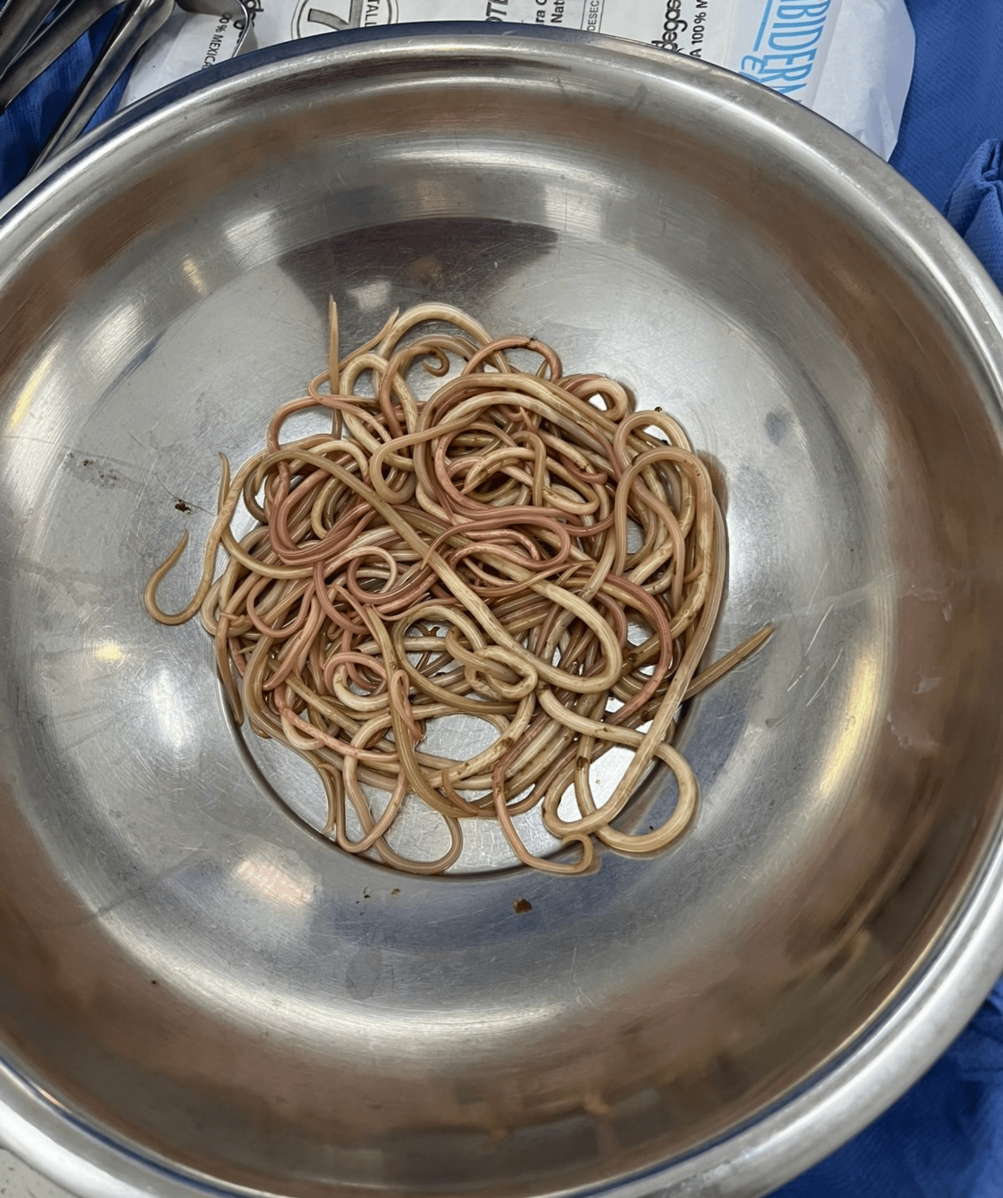
Gross appearance of multiple Ascaris lumbricoides worms extracted from the small intestine.

**Figure 3:**
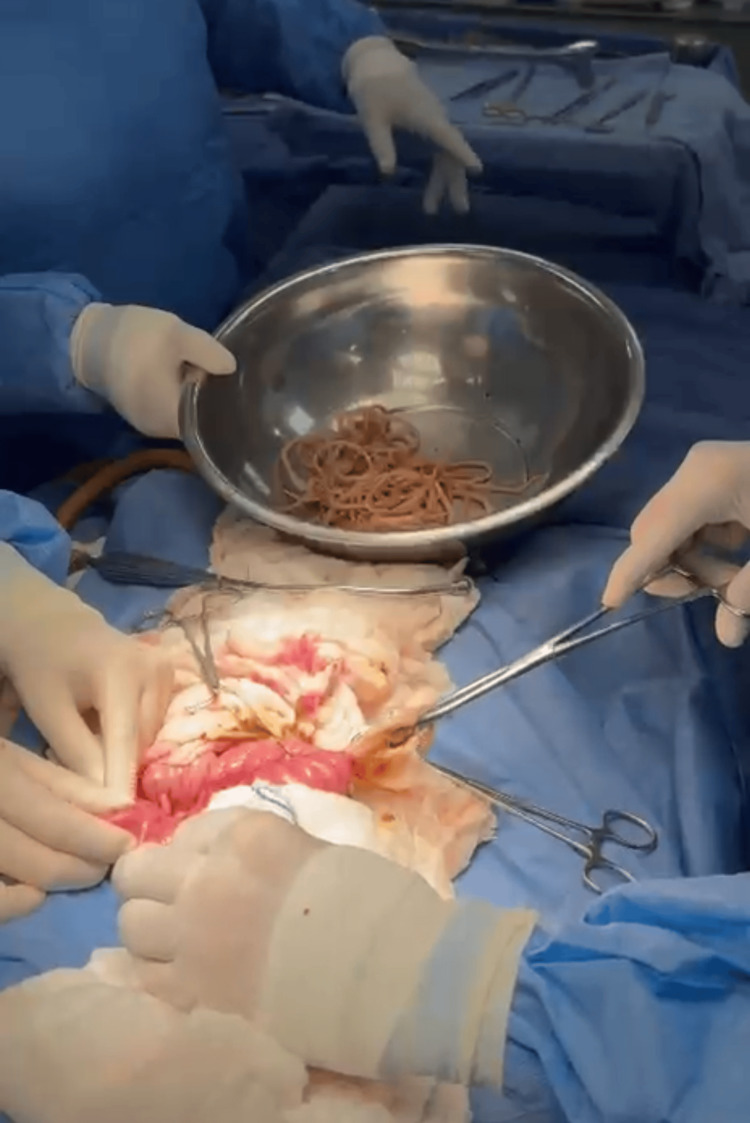
Intraoperative enterotomy with removal of Ascaris lumbricoides worms.

The patient was stabilized with intravenous fluids, antibiotics (ceftriaxone and metronidazole), and analgesia, and subsequently underwent emergency exploratory laparotomy. Upon entering the abdominal cavity, 500 mL of citrine peritoneal fluid was aspirated. A complete obstruction was identified approximately 40 cm distal to the ligament of Treitz, due to a large conglomerate of *A. lumbricoides*. An enterotomy was performed with careful removal of multiple worms until the lumen was completely cleared, followed by closure in two layers. A Penrose drain was placed in the pelvic cavity. The conglomerate of *A. lumbricoides* (round worms) extracted during surgery is shown in Figure 2, and the intraoperative enterotomy with removal of worms is illustrated in Figure 3.

The patient remained stable postoperatively. Oral intake was reintroduced progressively after bowel sounds returned on the third postoperative day. She was discharged in good condition with prescriptions for albendazole and ivermectin for deworming, along with outpatient follow-up.

## Discussion

Ascariasis continues to pose a substantial burden in low-resource settings and remains a major cause of pediatric intestinal obstruction [1,2]. Children are especially vulnerable due to environmental exposure and anatomical factors, and heavy infestation may result in mechanical obstruction. In our case, imaging demonstrated diffuse bowel dilation and free intraperitoneal fluid; however, the worm bolus was not visualized, consistent with reports noting that *Ascaris* aggregates frequently mimic fluid-filled bowel loops on CT and can remain radiologically occult [3-5].

Although eosinophilia is classically associated with helminthic infections, it may be absent in acute obstruction, particularly when inflammatory stressors, such as bacterial translocation, dehydration, or early peritoneal irritation, trigger a predominantly neutrophilic response [1]. 

Volvulus was initially considered due to the CT appearance of segmental narrowing. Colonoscopic decompression can be considered in selected cases of sigmoid volvulus [1]; however, in this setting, pediatric colonoscopy services were not available, and the patient exhibited signs of complete obstruction and peritoneal irritation. In such scenarios, immediate surgical exploration is recommended, especially in young children and in institutions without advanced endoscopic capabilities.

Intraoperative findings confirmed a dense worm bolus causing complete jejunal obstruction. When the parasite mass cannot be manually advanced into the colon, enterotomy remains the safest and most effective treatment, as supported by surgical experience in endemic regions [1-5]. Resection is typically reserved for cases with bowel necrosis or perforation. Our patient demonstrated an excellent postoperative recovery following enterotomy and antiparasitic therapy.

This case aligns with previously reported presentations, underscoring the diagnostic challenges posed by radiologically occult worm boluses and the need for prompt surgical intervention in complete obstruction. Although volvulus secondary to *Ascaris* remains rare, it has been described and warrants urgent evaluation [1]. Importantly, this case reflects true real-world conditions in resource-limited settings, where delays in advanced diagnostics or endoscopy often necessitate immediate operative management.

Ultimately, this case demonstrates a rare but serious complication of pediatric ascariasis and highlights the importance of considering *A. lumbricoides* in the differential diagnosis of intestinal obstruction in endemic regions. Strengthening school-based deworming programs, sanitation initiatives, and early educational interventions remains critical for reducing the burden of ascariasis and preventing severe surgical presentations.

## Conclusions

This case underscores a rare yet critical complication of pediatric ascariasis resulting in complete intestinal obstruction. Imaging may reveal obstruction but often fails to identify the underlying parasitic etiology, making timely surgical evaluation essential in resource-limited settings. Enterotomy remains the most effective treatment when the worm bolus cannot be advanced distally. Improving public health measures, particularly school-based deworming, sanitation programs, and early recognition of parasitic infections, continues to be key in preventing severe complications. Increased awareness of parasitic causes of intestinal obstruction may help clinicians reduce morbidity and mortality in endemic regions.
